# Evaluation of the Self-Healing Capacity of Asphalt Concrete with Polymer Capsules Containing Rejuvenator Under Various Cyclic Load Conditions

**DOI:** 10.3390/ma18225201

**Published:** 2025-11-17

**Authors:** Pei Wan, Zirong Ma, Zhiming Lin, Peixin Zhong, Xiaobin Zou, Yilun Shen, Niecheng Lin, Hang Chen, Jiazhu Wang, Shaopeng Wu, Quantao Liu, Lei Zhang, Xing Gong

**Affiliations:** 1 Fujian Provincial Transportation Research Institute Co., Ltd., Fuzhou 350004, China; wanpei@fjits.net (P.W.); mazirong@fjits.net (Z.M.); linzhiming@fjits.net (Z.L.); zhongpeixin@fjits.net (P.Z.); zouxiaobin@fjits.net (X.Z.); shenyilun@fjits.net (Y.S.); linniecheng@fjits.net (N.L.); 2 Superior College for Engineers, Chang’an University, Xi’an 710064, China; 3 State Key Laboratory of Silicate Materials for Architectures, Wuhan University of Technology, Wuhan 430070, China; wusp@whut.edu.cn (S.W.); liuqt@whut.edu.cn (Q.L.); 4 Wuhan University of Technology Advanced Engineering Technology Research Institute of Zhongshan City, Zhongshan 528400, China; 5 School of Materials Science and Engineering, Chang’an University, Xi’an 710064, China; lei.zhang@chd.edu.cn; 6 Research Institute of Highway Ministry of Transport, Beijing 100088, China; x.gong@rioh.cn

**Keywords:** asphalt concrete, self-healing capsule, healing level, load condition

## Abstract

Under the effect of cyclic load, calcium alginate (Ca-alginate) capsules can release the asphalt rejuvenator gradually, which provides asphalt concrete with a sustained healing ability during its service period. The rejuvenator release is significantly influenced by load cycles, pressure, and frequency, factors that have been overlooked in previous studies. To address this gap, this study investigates the self-healing performance of capsule-modified asphalt concrete under various cyclic load conditions. Calcium alginate capsules with rejuvenator are fabricated and characterized. The healing efficiency of concrete beams with capsules under different load patterns is evaluated. Additionally, the rejuvenator release rate from capsules after cyclic load is measured. The rheological behavior and the chemical composition of the extracted asphalt binder are also examined. Results show that the prepared capsules exhibit a multi-chamber structure and satisfy mechanical and thermal requirements. The healing ratio of specimen beams improves with increasing load cycles and pressure but decreases with higher load frequency. Under fixed load pressure (0.7 MPa) and frequency (1 Hz), the healing ratio of beams with capsules after 128,000 cycles of load can reach 75%. The rejuvenator is released gradually from the capsules. Under constant load cycles, the release ratio rises with greater load pressure but declines as load frequency increases. Under 64,000 cycles of load and 1 Hz of load frequency, the rejuvenator release ratio of capsules increases from 49.5% to 61.5% when the load pressure increases from 0.7 MPa to 1.40 MPa. The released rejuvenator enhances the flow ability of asphalt. Furthermore, it helps rebalance the chemical composition of asphalt by increasing the content of light components, thereby contributing to asphalt regeneration. This paper provides theoretical support for the service life of capsules under various traffic load conditions, facilitating their practical application and promotion in road engineering projects.

## 1. Introduction

Asphalt pavement pre-maintenance is an important task aimed at preventing pavement damage, prolonging the service lifespan of asphalt pavement, and keeping it in good service condition [1,2]. Among the existing road diseases, the prevention and control of cracking have emerged as the predominant issue in asphalt pavement maintenance strategies. At present, maintenance departments at home and abroad mainly utilize fog seal [3,4], micro-surfacing [5,6], thin layer [7,8], and other strategies to address pavement cracking. These interventions are passive repair after aging and cracks appear on the pavement, and the repair effect is unsatisfactory. Repeated crack repair initiatives lead to significant resource consumption and economic losses, while seriously affecting roadway capacity and traffic safety. Moreover, pavement cracks frequently occur in blind areas, which prevents traditional manual repair methods from being applied [9]. Current crack repair technologies are limited, necessitating the development of more advanced solutions.

Asphalt concrete demonstrates a demonstrable capability for autonomous healing of micro-cracks [10,11]. Utilizing this healing property to repair the micro-cracks in asphalt pavement in a budding state has become an advanced maintenance concept advocated by scholars at home and abroad. Although asphalt concrete possesses autonomous healing properties, the kinetics of the process are very slow, and its effectiveness is restricted to the partial recovery of small cracks. To improve the self-healing ability of asphalt concrete, scholars have developed four kinds of self-healing methods, such as induction heating, microwave heating, core–shell structure microcapsules, and multi-chamber capsules [11,12,13,14].

Induction heating self-healing technology functions by applying an alternating electromagnetic field to asphalt concrete mixed with conductive materials, which causes the asphalt mastic to rapidly warm up and expand and flow to the micro-cracks, thus prompting the micro-cracks to close [15,16,17]. This technology could bring high crack repair efficiency and multiple repair times for asphalt concrete. However, the induction heating technique is characterized by a gradient heating effect, which confines its repair efficacy to surface-layer micro-cracks within a depth of 4 cm [18,19]. Meanwhile, it does not reinstate the original rheological and chemical properties of the aged asphalt, resulting in cracks developing quickly in the healed pavement. Additionally, the lack of large-scale induction heating units limits its actual application.

Microwave-induced heating self-healing technology functions through the addition of microwave-absorbing materials in asphalt concrete, and then with the help of microwave irradiation to make the asphalt concrete’s temperature rise. The viscosity of the binder surrounding the crack reduces, and the rates of capillary flow and molecular diffusion improve, thus achieving the effect of crack repair [20,21,22]. Microwave heating is uniform and has a large heating and healing depth. However, long-term microwave irradiation may damage the chemical structure of asphalt molecules, accelerating the aging and performance decay of asphalt [23], and is not suitable for promoting its application in asphalt pavement.

Microcapsule-based self-healing technology involves incorporating core–shell capsules containing rejuvenator into asphalt concrete; upon the crack reaching the microcapsule, the capsule shell ruptures to release the repair agent, and the agent fills in the crack to prevent its further expansion, thus realizing the self-healing of cracks [24,25]. The microcapsules are fabricated using in situ polymerization, featuring a polymer shell and an asphalt rejuvenator core [26,27,28,29]. This technology has a certain crack healing effect, but it requires microcapsule rupture to release the healing agent. Additionally, the matching difficulty between crack tip stress and capsule strength results in the uncontrollable rupture behavior of the microcapsule.

To improve the encapsulation of the repair agent and the repair effect, researchers encapsulated the asphalt rejuvenator into millimeter-sized multi-chamber capsules, expecting the capsules to gradually release the repair agent from the different chambers under the action of driving loads, thus conferring to the asphalt concrete long-term self-healing performance. Micaelo et al. developed millimeter-scale calcium alginate capsules through ionic cross-linking. Their results demonstrate that these capsules deform and rupture under mechanical stress, thus releasing the healing agent and enhancing the healing ability of the asphalt [30]. Al-Mansoori et al. prepared the same multi-chamber calcium alginate capsule and found that the capsule has good thermal stability, the rupture rate of the capsule is less than 2% after asphalt mixture crushing and molding, and the incorporation of the capsule does not degrade the road performance of asphalt concrete. After applying 0.6 MPa/350 times of cyclic load, the rupture rate of the capsules reaches more than 80%, and the healing rate of asphalt concrete reaches 95% [31,32,33]. Liu et al. adjusted the internal microstructure of the calcium alginate capsules to enhance their stress-response behavior. It was found that capsules with a micro-nano-chamber architecture can realize the gradual release of the encapsulated healing agent inside through elastic deformation without rupturing under cyclic load, thereby significantly improving the healing rate of asphalt concrete [34,35,36,37]. Compared with the traditional microcapsule with core–shell structure, multi-chamber calcium alginate capsules offer high healing agent content, efficient repair performance, and sustained effectiveness. Under traffic load, the capsules autonomously heal micro-cracks and rejuvenate aged asphalt, which is regarded as a kind of asphalt concrete self-healing technology with great development potential.

The release of healing agent from the calcium alginate capsules depends on the applied cyclic load, and the load cycle, pressure, and frequency may determine the rejuvenator release by the capsule, which in turn affects the healing efficiency of asphalt concrete. However, current research only focused on the healing outcome of asphalt concrete with calcium alginate capsules under fixed load conditions and has not yet considered the healing effect under different load conditions. In the actual service process of the pavement, the traffic volume on the pavement, the tire pressure, and speed of vehicles influence the action behavior of capsules in asphalt concrete, thereby regulating the release of the rejuvenator and the overall repair effectiveness.

**Figure 1:**
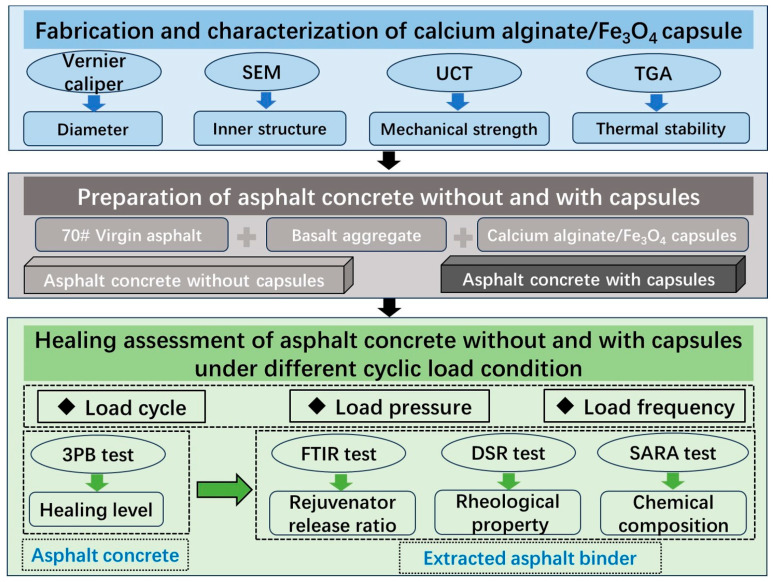
The flowchart of this research.

Therefore, this study evaluates the healing properties of asphalt concrete with calcium alginate capsules under varying load patterns, including load cycles, pressure, and frequency. The calcium alginate/Fe_3_O_4_ capsules are synthesized in this work, and their key material properties are systematically evaluated. The healing efficiency of capsule-modified asphalt under various load conditions is evaluated via a fracture–healing–refracture protocol. The rejuvenator release ratio is quantified using FTIR, while the rheological and chemical properties of the extracted binder are assessed via DSR and TLC-FID, respectively. The whole research flowchart is presented in Figure 1.

## 2. Materials and Methods

### 2.1. Raw Materials

The capsules are fabricated using sodium alginate, nano Fe_3_O_4_, asphalt rejuvenator, Tween 80, anhydrous calcium chloride, and tap water as the primary components. Table 1 presents the key properties of the raw materials. Sunflower oil is used to regenerate the aged asphalt by supplying the absent light components [38,39]. Moreover, the FTIR spectrum of sunflower oil displays an obvious absorption peak at 1745 cm^−1^, while the base 60/80 asphalt has no absorption peak within this range [40,41,42]. The basic information of sunflower oil is presented in Table 2.

### 2.2. Preparation of Ca-Alginate/Fe_3_O_4_ Capsules via Ionic Gelation

**Figure 2:**
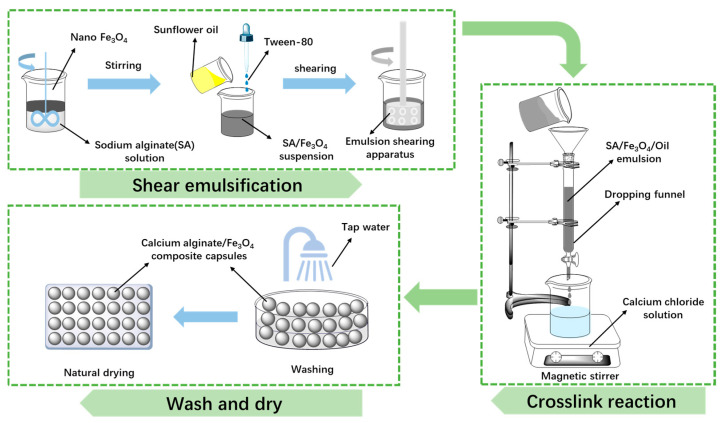
The fabrication procedure of calcium alginate/Fe_3_O_4_ capsules [43].

The preparation of Ca-alginate/Fe_3_O_4_ capsules comprises three main steps (as shown in Figure 2). Initially, a stable emulsion is formed by homogenizing nano-Fe_3_O_4_ powder, Tween 80, and sunflower oil within a sodium alginate solution under shear emulsification (shear rate: 5000 rpm, shear time: 15 min). Subsequently, the emulsion is dripped via a separating funnel into a calcium chloride solution, inducing ionic cross-linking to form capsules. Lastly, the capsules are rinsed thoroughly with tap water and air-dried under ambient conditions. The detailed steps and conditions of this fabrication process have been previously described in relevant research [43,44,45].

### 2.3. Performance Evaluation of Capsules

A series of tests are conducted to assess the key properties of the fabricated capsules. Since the diameter of the prepared calcium alginate capsules exceeded 1000 μm, it was not possible to measure the diameter of the capsules by laser measurement. Therefore, in this paper, the diameters of 50 randomly selected capsules were manually measured using vernier calipers. The mean value was selected as the diameter of capsule. The external morphology of the capsules was documented with a digital camera, while their internal structure was characterized using a scanning electron microscope (Gemini 300, Zeiss, Jena, Germany). The mechanical strength of the capsule was determined via a uniaxial compression test by a universal material test machine (5967, Instron, Norwood, MA, America). The load application speed was 0.5 mm/min. The thermal resistance of the capsule was explored by the simultaneous thermal analyzer (STA449F3, Netzsch, Selb, Germany). The tests were carried out at a heating rate of 10 °C/min, with the temperature ranging from 40 °C to 990 °C. The atmosphere was N_2_ and the inject rate was 20 mL/min.

### 2.4. Preparation of Asphalt Mixtures Incorporating Capsules

**Figure 3:**
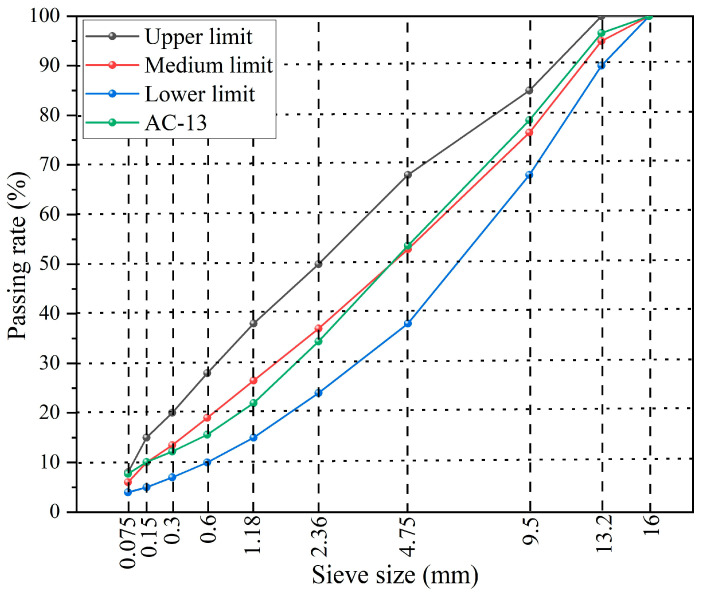
The AC-13 gradation curve used in this work.

In this study, a dense-graded AC-13 asphalt mixture is utilized, with its gradation provided in Figure 3. The capsules are incorporated into the mixtures at an optimum content of 0.5% by mass during mixing. Subsequently, asphalt concrete plates, both with and without capsules, are produced using a rutting plate compactor. The asphalt concrete beams measuring 98 mm × 45 mm × 50 mm are extracted. A notch of 5 mm × 4 mm is carefully created in the center of each beam.

### 2.5. Healing Assessment of Asphalt Concrete Under Different Cyclic Load Conditions

**Figure 4:**
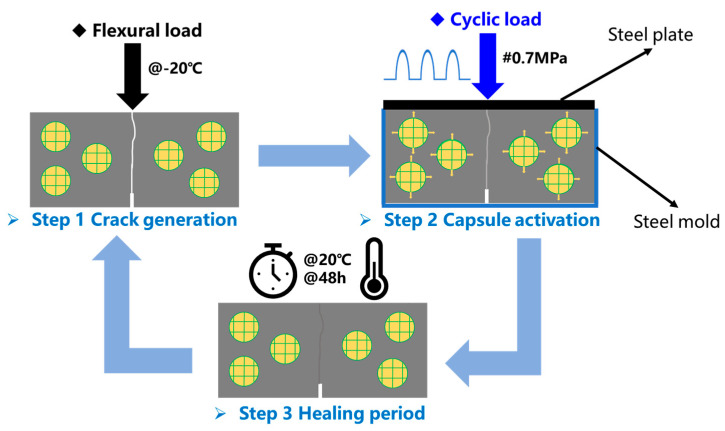
The cyclic load–healing test procedure.

A fracture–cyclic load–healing–refracture procedure is performed on normal asphalt concrete beams and capsules modified beams, following the four-step process illustrated in Figure 4. Initially, a three-point bending (3 PB) test is carried out at −20 °C with a load rate of 0.5 mm/min to introduce cracking and determine the initial flexural strength. Subsequently, the cracked beams are placed into a steel mold covered with a steel plate and then subjected to cyclic load to apply uniform compressive stress. Afterward, the beams are transferred to a temperature-controlled chamber and healed at 20 °C for 48 h. Finally, a second 3 PB test is conducted on the healed beams under the same conditions to evaluate their recovered strength. Each experimental group is replicated three times in parallel. The average of the three trial results was taken as the final experimental result.

The number of fatigue loads in the fracture–healing tests used in this paper is set according to the design parameters of medium traffic grade pavements. The parameters in Table 3 represent only a part of the number of loads acting on each point of the pavement cross-section, and the specific values need to be calculated according to the cumulative equivalent axle times of the design traffic volume of the pavement, lane coefficients, wheel-mill distribution coefficients, and the equivalent small car axle load conversion factors. The cumulative number of loads on the capsule during the design life of a medium traffic grade pavement is calculated to be 240,000. Therefore, the number of loads per year on the actual pavement cross-section at various points during the specified design service life is 16,000. In this paper, the numbers of cyclic loads are set to 0, 16,000, 32,000, 64,000, 96,000, 128,000, and 160,000 times, which simulates the cumulative numbers of load on the capsule on the cross-section of the pavement in the actual pavement load of 0, 1, 2, 4, 6, and 8 years, respectively.

With the booming development of transportation, the volume of road traffic has gradually increased, and the axle load of vehicles has also increased, especially the axle load and tire pressure of heavy trucks have also increased; therefore, there is a large discrepancy between the actual tire pressure on the road and the standard tire pressure (0.70 MPa). Therefore, cyclic loads with different pressures (0.70 MPa, 1.05 MPa, and 1.40 MPa) are applied on the asphalt concrete beam to investigate its healing level under different simulated vehicle tire pressures.

In the actual pavement service process, the speed of traveling vehicles affects the load time of traveling loads on asphalt pavements, which in turn affects the frequency of load application. Relevant studies show that the repeated load frequencies of 5 Hz, 10 Hz, and 15 Hz (converted to pavement load times of 0.2 s, 0.1 s, and 1/15 s, respectively) are equivalent to the actual driving speed of road vehicles at 30 km/h, 60 km/h, and 90 km/h, as shown in Table 4.

The strength recovery ratio (H_S_) and fracture energy recovery ratio (H_E_) are the selected healing index to evaluate the healing performance of the asphalt beam after cyclic load.

(1) $$H_{S}=\frac{F_{1}}{F_{0}}$$

(2) $$H_{E}=\frac{E_{1}}{E_{0}}$$

where F_0_ and F_1_ denote the bending strength before and after healing, respectively, while E_0_ and E_1_ represent the corresponding fracture energy.

### 2.6. Characterization of Rejuvenator Release from Capsules in Asphalt Concrete After Cyclic Load

The FTIR spectrum of sunflower oil exhibits a distinct absorption peak (1745 cm^−1^), which is absent in the base 60/80 asphalt. This unique spectral feature enables the quantification of the rejuvenator released from capsules into the asphalt concrete after cyclic load using FTIR spectroscopy. The specific procedure is as follows:

(1) The relation establishment between absorption peak index I_1745cm^−1^_ and oil concentration in asphalt

Firstly, different dosages (0, 2%, 4%, 6%, 8% of asphalt mass) of oil are added into the asphalt and then the mixtures are blended carefully for 30 min at 130 °C. Secondly, the asphalt blends with different oil dosages are characterized by FTIR spectroscopy, employing a spectral range of 400–4000 cm^−1^. The test is conducted with a resolution of 4 cm^−1^ and a total scanning duration of 64. Thirdly, the FTIR spectrum of asphalt specimen is analyzed through OMNIC software (Version 9.0). As demonstrated in prior research, the spectral index at 1745 cm^−1^ (Equation (3)) enables the quantification of oil content within asphalt binders [46].

(3) $$I_{{1745cm}^{-1}}=\frac{The\;peak\;area\;of\;{1745\;cm}^{-1}}{\sum Area\;of\;spectral\;bands\;between\;2000\;and\;600\;{cm}^{-1}}$$

**Figure 5:**
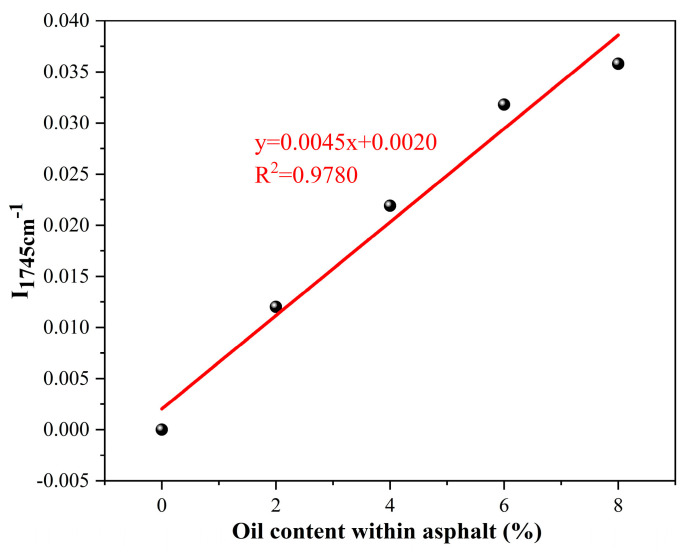
The relationship between I_1745cm^−1^_ and oil content within asphalt [46].

The relationship between I_1745cm^−1^_ and the oil concentration of the asphalt binder is shown in Figure 5. The I_1745cm^−1^_ index exhibited a linear upward trend as the oil concentration in the asphalt binder increased (y = 0.0045x + 0.0020, R^2^ = 0.978). Therefore, the oil release ratio of capsules after cyclic load is determined via the I_1745cm^−1^_ of extracted asphalt binder.

(2) The extraction of asphalt from asphalt concrete incorporating capsules after load

**Figure 6:**
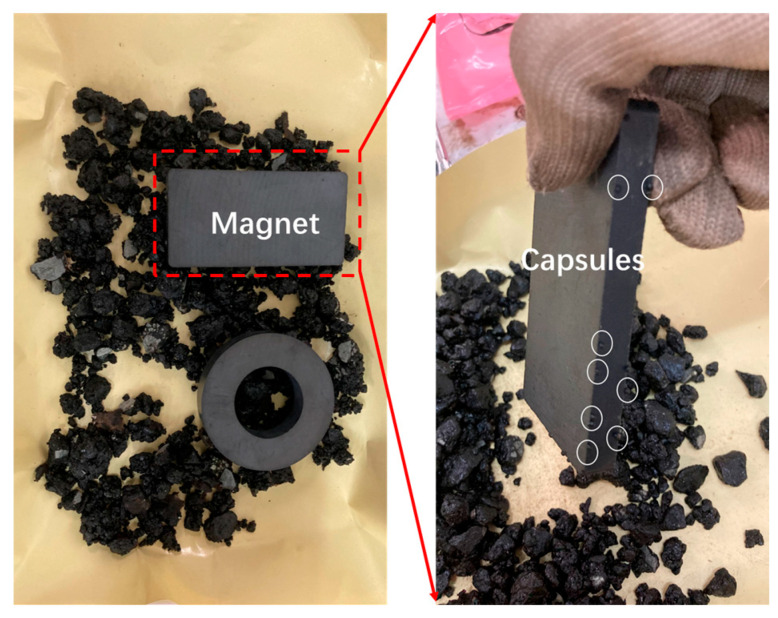
The magnetic separation of capsules within loose asphalt mixtures.

The beams are placed in an oven maintained at 80 °C for 45 min. The capsules within the loose mixture are subsequently removed via employing a combination of magnetic attraction and manual techniques, as illustrated in Figure 6. Subsequently, the remaining asphalt mixtures are dissolved in trichloroethylene for 48 h. The supernatant is collected and allowed to evaporate in a fume hood for 24 h. Subsequently, 0.1 g of extracted asphalt binder is dissolved in 2 mL of CS_2_ within a centrifuge tube. A portion of the resulting solution is then drop-cast onto KBr pellets and dried to form thin films for FTIR analysis. Each experimental group is replicated three times in parallel. The average of the three trial results was taken as the final experimental result.

### 2.7. Rheological Characteristics of Asphalt Binder Under Different Load Scenarios

Vegetable oil improves the flow properties of asphalt binder, thereby enhancing its low-temperature crack resistance. To evaluate the rheological behavior of the binder after cyclic load, an oscillatory temperature sweep test is performed using a dynamic shear rheometer (Smartpave 102, Anton Paar, Graz, Austria). The test ranges from −10 °C to 30 °C using an 8 mm rotor, at a fixed strain of 0.05% and a frequency of 10 rad/s. Each experimental group is replicated three times in parallel. The average of the three trial results was taken as the final experimental result.

### 2.8. Chemical Composition Characteristics of Extracted Asphalt Binder After Various Cyclic Load Treatments

The regeneration effect of released oil on asphalt is investigated from the asphalt composition dimension. The relative contents of saturate, aromatic, resin, and asphaltene fractions of the extracted asphalt samples are determined using thin-layer chromatography–hydrogen flame ion detector (TLC-FID, Tokyo, Japan). The experiment utilized N_2_ flow rates of 2.0 L/min and 160 mL/min, respectively, and a scanning speed of 30 s per acquisition. Three technical replicates are measured for each asphalt sample. Each experimental group is replicated three times in parallel. The average of the three trial results was taken as the final experimental result.

## 3. Results and Discussion

### 3.1. Basic Performance of Capsules

**Figure 7:**
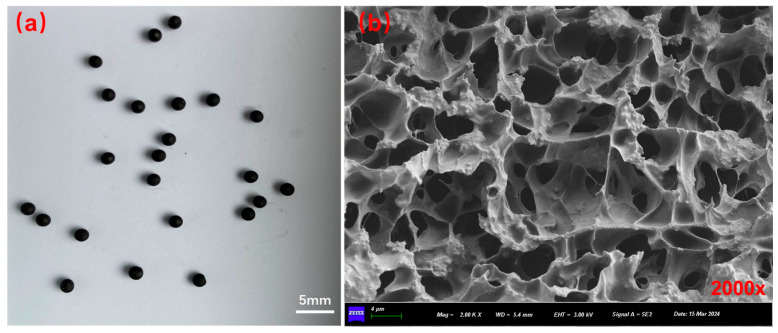
Macroscopic morphology (**a**) and internal structure (**b**) of capsules [48].

The morphological structure of capsules is presented in Figure 7. The basic information of the capsules is summarized in Table 5. The outer appearance of capsules shows a sphere shape, and the capsules have a multi-chamber structure. The capsules, with an average diameter of 1.8 mm, can be integrated into asphalt mixtures to partially replace fine aggregates. The mechanical strength of the capsules is 11.8 N, which satisfies the requirement (10 > N) of aggregate type capsules in asphalt concrete preparation [47]. Additionally, the capsules show a mass loss of only 3.9% at 200 °C, demonstrating sufficient thermal stability to withstand high-temperature asphalt mixing processes.

### 3.2. Assessment of Asphalt Concrete Self-Healing Under Various Cyclic Load Conditions

#### 3.2.1. Healing Performance of Test Beams After Various Load Cycles

**Figure 8:**
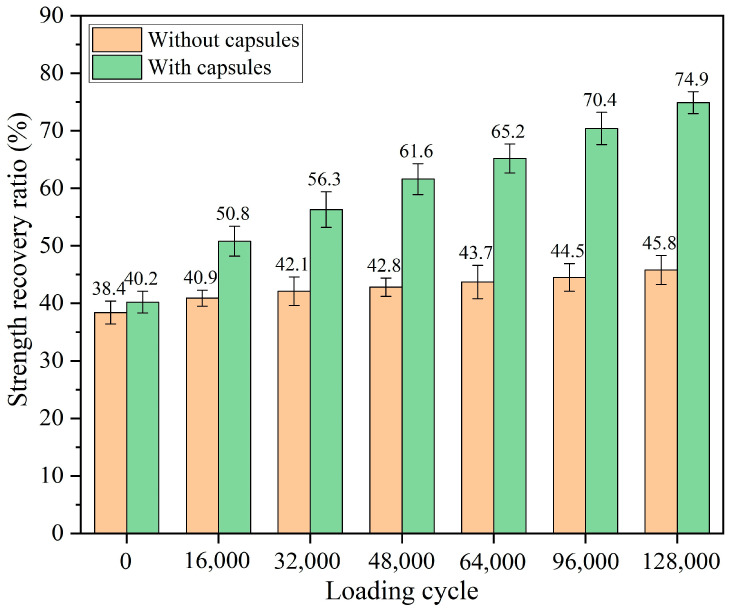
Strength recovery ratio of beams under various load cycles [48].

**Figure 9:**
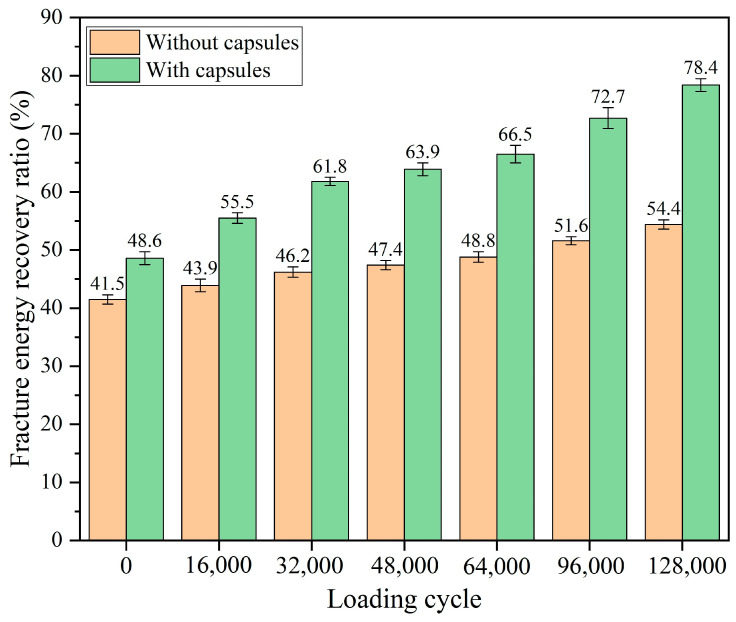
Facture energy recovery ratio of beams under various load cycles.

The recovery rates for both strength and fracture energy under different numbers of load cycles are presented in Figure 8 and Figure 9, respectively, where no capsule indicates ordinary asphalt concrete without capsules and capsule indicates asphalt concrete with capsules. After 0, 16,000, 32,000, 48,000, 64,000, 96,000, and 128,000 cycles of load, the strength recovery rates of ordinary asphalt concrete are 38.4%, 40.9%, 42.1%, 42.8%, 43.7%, 44.5%, and 45.8%, respectively, and the fracture recovery rates of ordinary asphalt concrete are 41.5%, 43.9%, 46.2%, 47.4%, 48.8%, 51.6%, and 54.4%, respectively. As a kind of viscoelastic material, asphalt has certain inherent repair characteristics; therefore in the absence of any external load conditions, the strength recovery rate of asphalt concrete could be up to 38%, and the fracture energy recovery rate could be up to 41%. With the increase in load cycle, asphalt concrete beams without capsules gradually become dense under the extrusion of load, the degree of embedding between the aggregates deepens, the bond between the asphalt is tighter, and the fracture surfaces of the concrete are closer to each other under the extrusion; therefore the healing ratio of ordinary beams gradually improved with increasing load cycles.

After 0, 16,000, 32,000, 48,000, 64,000, 96,000, and 128,000 load cycles, the strength recovery rates of the beam with capsules are 40.2%, 50.8%, 56.3%, 61.6%, 65.2%, 70.4%, and 74.9%, respectively, and the fracture energy recovery rates of the beam with capsules are 48.6%, 55.5%, 61.8%, 63.9%, 66.5%, 72.7%, and 78.4%, respectively. Without cyclic load, the healing rate of the beam with capsules is slightly higher than that of the ordinary beam, which is due to the release of a small amount of sunflower oil from the capsules in the mixing and compaction process of the asphalt mixture in advance; the low-viscosity sunflower oil slightly softened the asphalt, which enhances the intrinsic repair potential of the asphalt. In addition, the healing rate of beams containing capsules is still at a low level after 48 h of repair period without external load. This indicates that external load is required to trigger the release of the sunflower oil from the capsules, thereby enhancing the self-healing ability of the asphalt concrete.

The healing capacity of capsule-modified beams is markedly superior to that of ordinary beams under identical cyclic load, thus confirming that capsule incorporation can substantially improve the self-healing performance of asphalt concrete. An improvement in the healing level is observed in the beams containing capsules as the load cycles increase. On the one hand, the intrinsic repair characteristics of asphalt itself and the denser asphalt concrete in the process of cyclic load application are beneficial to the improvement of the healing ratio. On the other hand, under the cumulative action of external cyclic load, the capsules within the asphalt concrete gradually release sunflower oil. The oil then diffuses into the surrounding cracked areas, thus softening the aged asphalt and reducing its viscosity. This process enhances asphalt mobility, facilitates crack closure, and contributes to the recovery of mechanical properties.

#### 3.2.2. Healing Performance of Test Beams Under Different Load Pressures

**Figure 10:**
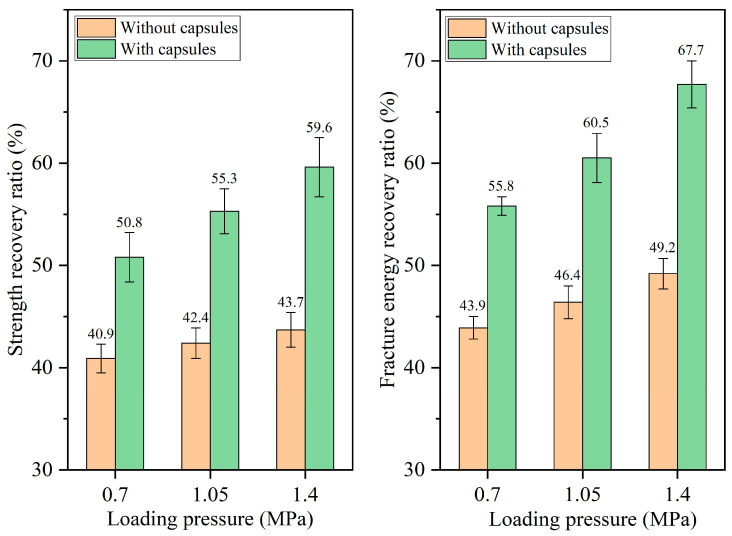
Healing level of beams under 16,000 load cycles at different load pressures.

**Figure 11:**
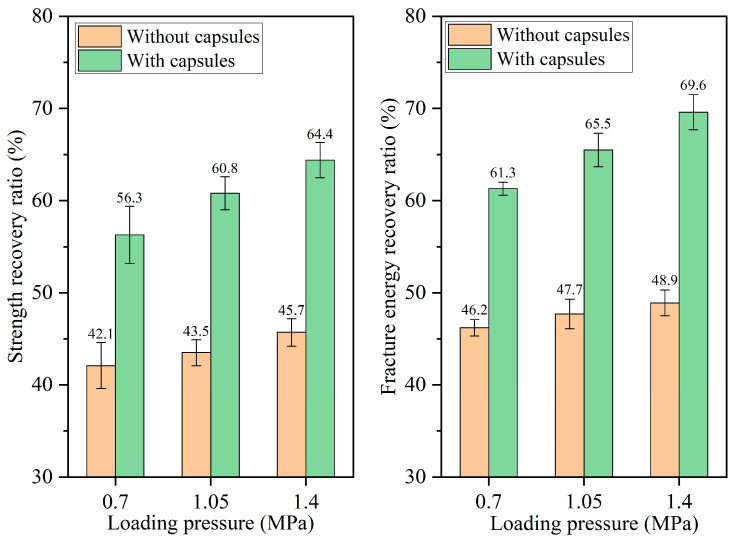
Healing level of beams under 32,000 load cycles at different load pressures.

**Figure 12:**
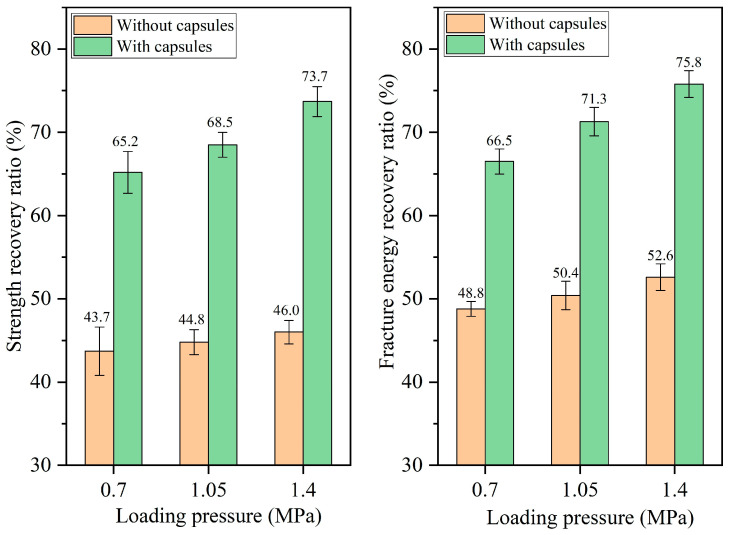
Healing level of beams under 64,000 load cycles at different load pressures.

**Figure 13:**
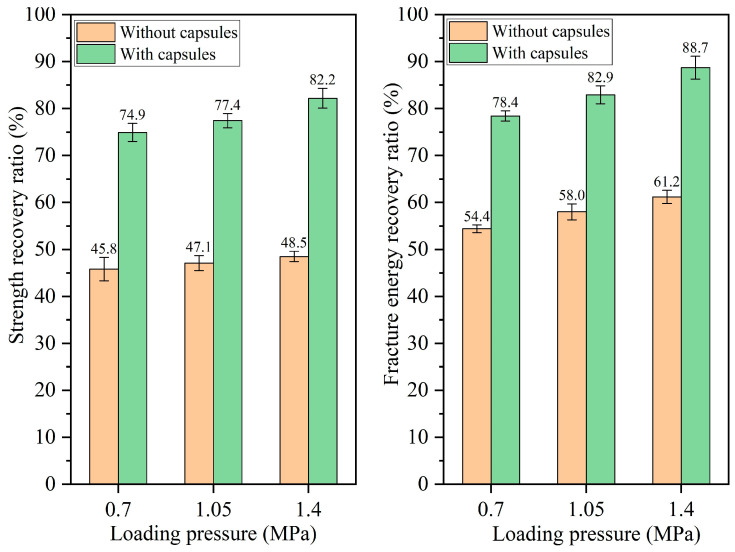
Healing level of beams under 128,000 load cycles at different load pressures.

The healing ratios of asphalt concrete under different load pressures at 16,000, 32,000, 64,000, and 128,000 cycles of load are presented in Figure 10, Figure 11, Figure 12, and Figure 13, respectively. When the number of loads is the same, the healing ratio of the ordinary beam improves slightly with the increase in load pressure. For example, under 64,000 load cycles, the strength recovery ratios of the ordinary beam under 0.7 MPa, 1.05 MPa, and 1.40 MPa are 43.7%, 44.8%, and 46.0%, respectively, and the corresponding fracture energy recovery rates of the ordinary beam are 48.8%, 50.4%, and 52.6%, respectively. Higher load pressure could make the fractured asphalt concrete denser, and the fracture surfaces in the concrete got closer together, thus slightly increasing the healing ratio of the beams.

The incorporation of capsules significantly enhances the healing rates of the beams under all three load pressures. Moreover, under a fixed load cycle, the healing rates of capsule-modified beams rise with elevated load pressure. When the load cycle is 128,000, the strength recovery rates of the beam under 0.70 MPa, 1.05 MPa, and 1.40 MPa are 74.9%, 77.4%, and 82.2%, respectively, and the fracture energy recovery ratios are 78.4%, 82.9%, and 88.7%, respectively. This result is attributed to the greater release of sunflower oil from the capsules under higher pressure, which enhanced the softening of the asphalt, accelerated its flow and diffusion around cracks, and thereby improved both the crack closure rate and the recovery of mechanical properties.

#### 3.2.3. Healing Performance of Beams Following Different Load Frequencies

**Figure 14:**
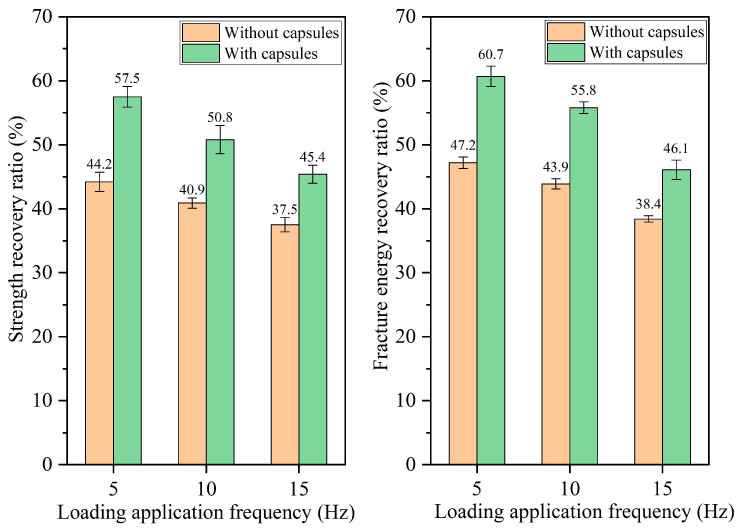
Healing level of beams under 16,000 load cycles at different load application frequencies.

**Figure 15:**
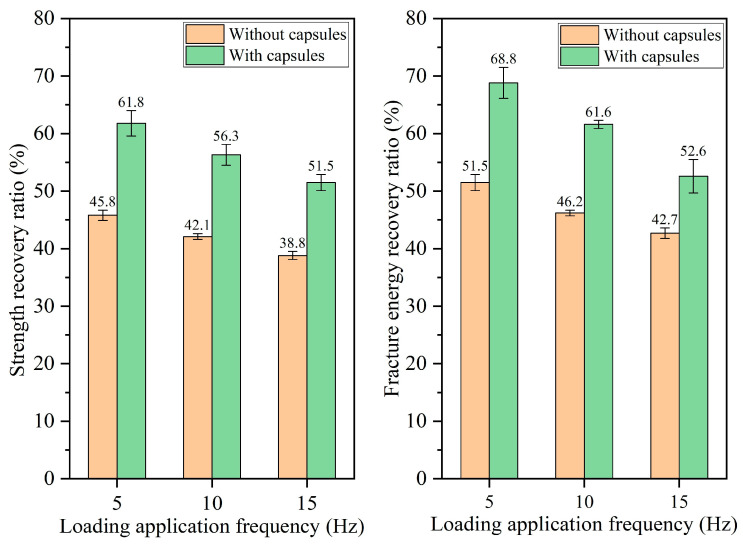
Healing level of beams under 32,000 load cycles at different load application frequencies.

**Figure 16:**
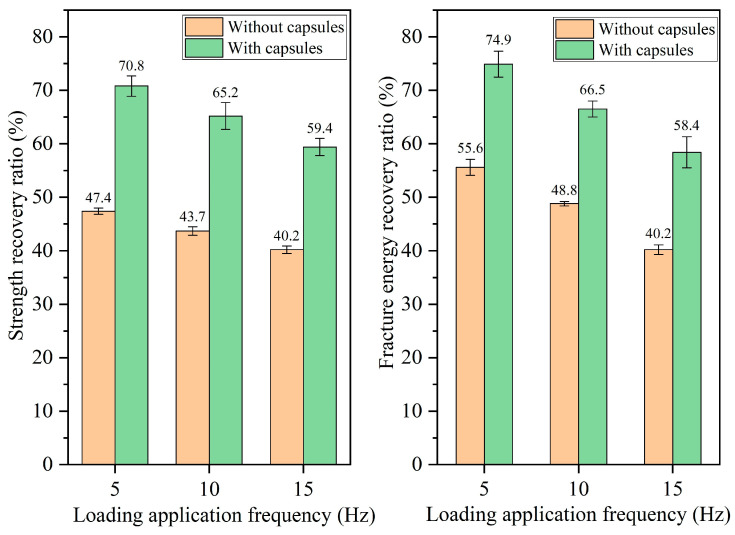
Healing level of beams under 64,000 load cycles at different load application frequencies.

**Figure 17:**
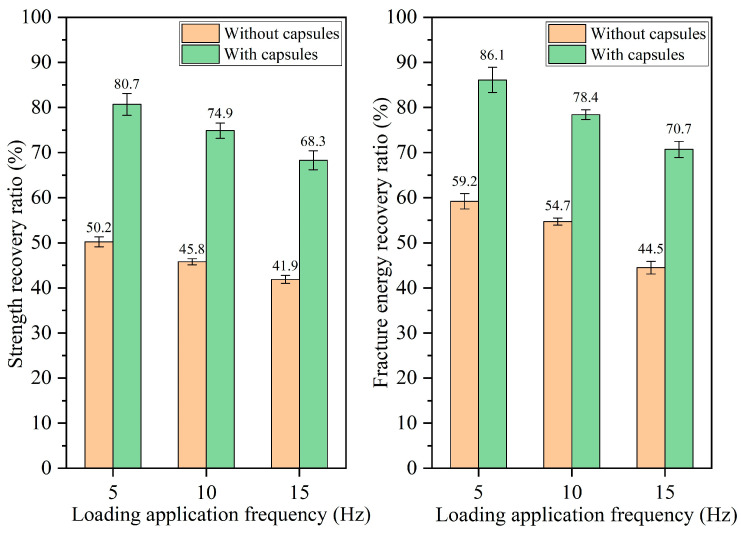
Healing level of beams under 128,000 load cycles at different load application frequencies.

The healing rates of beams under different load frequencies are presented in Figure 14, Figure 15, Figure 16 and Figure 17. For ordinary beams, the healing rates decrease with the increase in the load frequency under a fixed load cycle. For example, when the load cycle is 32,000, the strength recovery rates of ordinary asphalt concrete under load frequencies of 5 Hz, 10 Hz, and 15 Hz are 45.8%, 42.1%, and 38.8%, respectively, and the fracture energy recovery rate are 51.5%, 46.2%, and 42.7%, respectively. Under fixed load cycle, the higher load frequency leads to a shorter load time in the cyclic load and a lower total load time for the asphalt concrete beam, which indicates the compaction time of fractured asphalt concrete is shorter. Therefore, the healing effect is relatively poor.

When the capsule is incorporated into asphalt concrete, regardless of the load frequency, the capsule-embedded beams exhibit superior healing rates compared to ordinary beams under identical load conditions, demonstrating that variations in vehicle speed can activate the capsules and enhance the self-healing capacity of cracked beams. At a constant number of load cycles, the healing efficiency of asphalt concrete declines at higher frequencies. For example, when the number of loads is 128,000, the strength recovery rates of capsule-modified beams under different load frequencies of 5 Hz, 10 Hz, and 15 Hz are 80.7%, 74.9%, and 68.3%, respectively, and the corresponding fracture energy recovery rates are 86.1%, 78.4%, and 70.7%, respectively. Under a fixed load cycle, higher load frequency leads to lower total load time of beams in cyclic load and makes the load action time on the capsules become shorter, which will make the capsules release less rejuvenator and thus reduces the healing ratio of capsule-modified beams.

### 3.3. Rejuvenator Release Evaluation of Capsules After Cyclic Load

#### 3.3.1. Rejuvenator Release Ratio of Capsules After Different Cycles of Load

**Figure 18:**
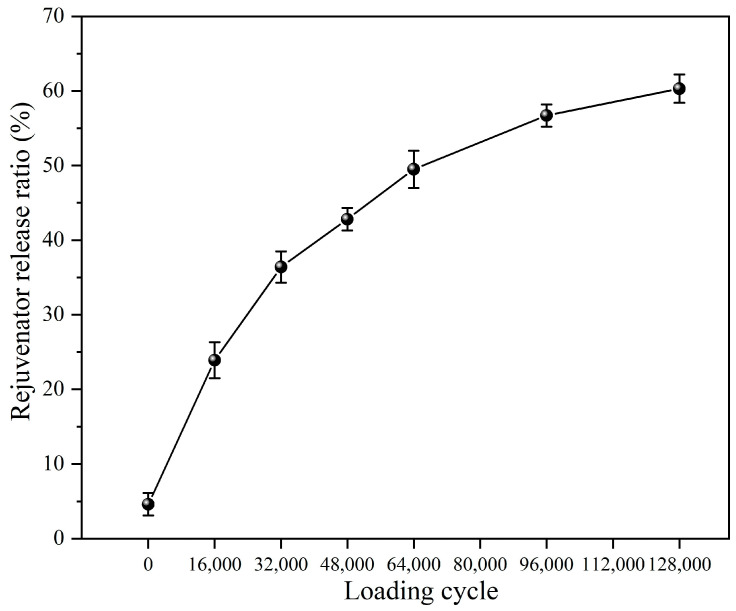
Rejuvenator release ratio of capsules after different cycles of load [48].

Figure 18 depicts the release ratio of the rejuvenator from capsules embedded in beams after varying cycles of load. The rejuvenator release ratios are 23.9%, 36.4%, 42.8%, 49.5%, 56.7%, and 60.3% after the asphalt concrete undergoes 16,000, 32,000, 48,000, 64,000, 96,000, and 128,000 load cycles, demonstrating a clear upward trend. As the load cycle increases, the response time of the capsules increases and the internal chamber extrusion time also increases, which improves the rejuvenator release ratio. The load–response release characterization of the capsules shows that the multi-chamber capsules have the ability of a long-lasting sustained release feature. In the cyclic load process, calcium–alginate composite capsules gradually release inner rejuvenator rather than releasing all the encapsulated rejuvenator at once, thus promoting sustainable healing in the asphalt concrete.

#### 3.3.2. Rejuvenator Release Ratio of Capsules Following Different Load Pressures

**Figure 19:**
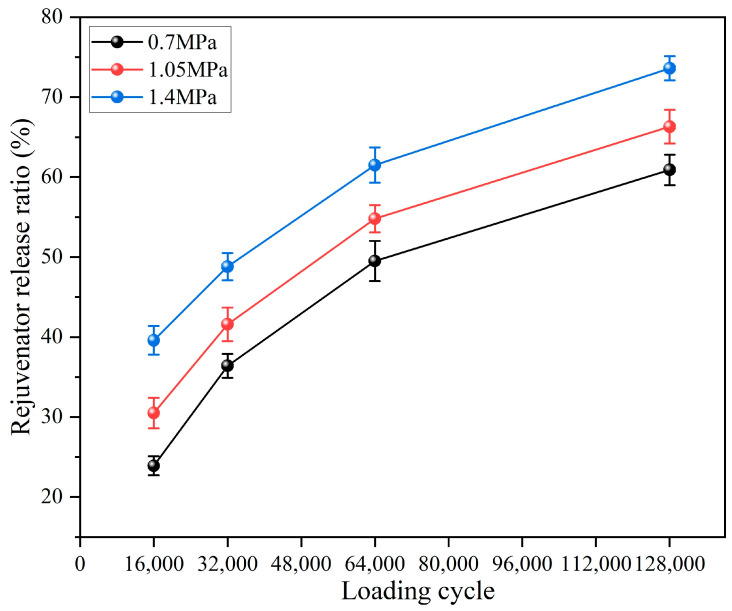
Rejuvenator release ratio of capsules after different load pressures.

The rejuvenator release features of capsules in asphalt concrete under different load pressures are shown in Figure 19. In general, under three types of load pressure, the rejuvenator release ratio increases with the load cycle, which indicates that the capsules in beams still present a sustained release feature under different load pressures. A positive correlation is observed between the rejuvenator release ratio and the applied load pressure under a constant number of load cycles. Specifically, under 32,000 cycles of load, the release ratio increases from 36.4% to 48.8% as the pressure rises from 0.70 MPa to 1.40 MPa. This suggests that higher vehicle tire pressures, under equivalent traffic volume, will promote a more substantial release of the rejuvenator. Overloading accelerates the release of rejuvenating agents, which may expedite crack closure and repair, thereby mitigating damage to asphalt pavements under severe traffic loading conditions.

#### 3.3.3. Rejuvenator Release Ratio of Capsules Following Different Load Application Frequencies

**Figure 20:**
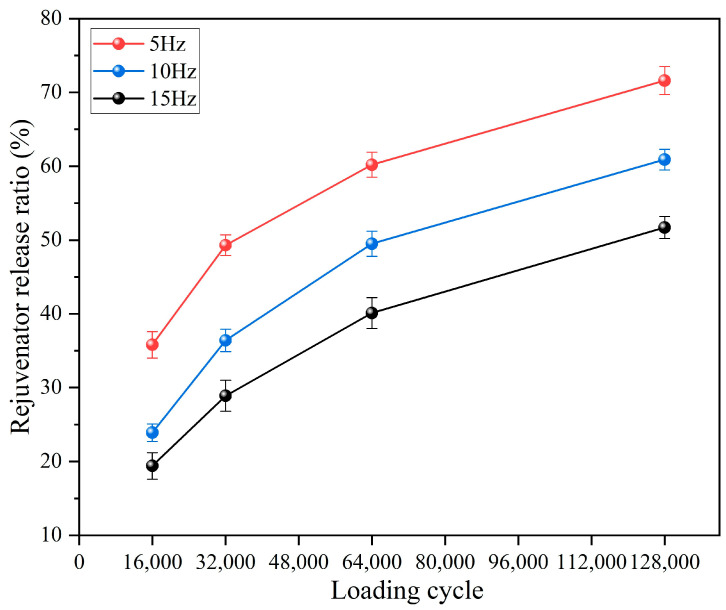
Rejuvenator release ratio of capsules after different load frequencies.

As shown in Figure 20, the rejuvenator release ratio consistently increases with the number of load cycles under all three tested frequencies. This indicates that capsule activation and healing agent release occur under vehicular load irrespective of traffic speed, ensuring functionality throughout the pavement’s service life. Under a fixed load cycle, the rejuvenator release ratios reduce as the load frequency application increases. For example, under 64,000 cycles of load, the rejuvenator release ratios are 60.2%, 49.5%, and 40.1% at load application frequency of 5 Hz, 10 Hz, and 15 Hz, respectively. When the total load cycles are unchanged, higher load frequency leads to shorter single load action time, which means that the total stress action time on capsules will reduce and the released rejuvenator will decrease. Excessive speed accelerates the performance degradation of asphalt pavements, while the release of rejuvenators within capsules may slow the deterioration of the service performance.

### 3.4. Rheological Characteristics of Asphalt Binder Following Cyclic Load

#### 3.4.1. Rheological Property of Asphalt Under Different Cycles of Load

**Figure 21:**
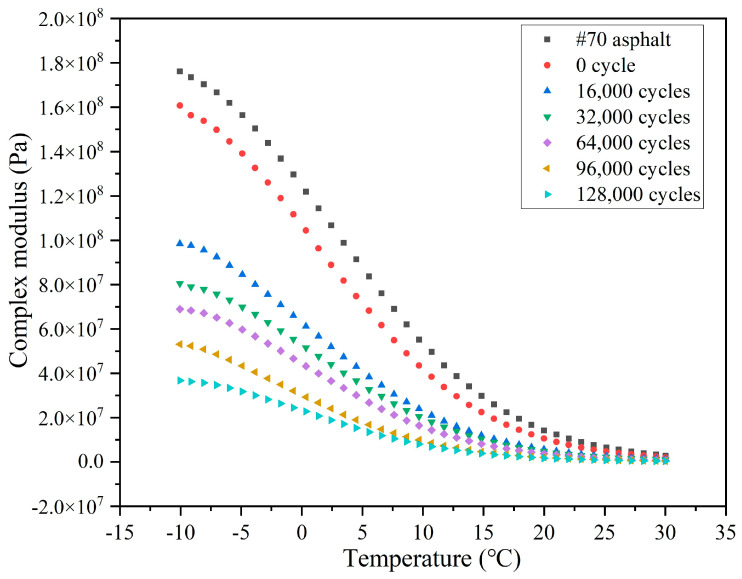
The G* of asphalt after different cycles of load.

**Figure 22:**
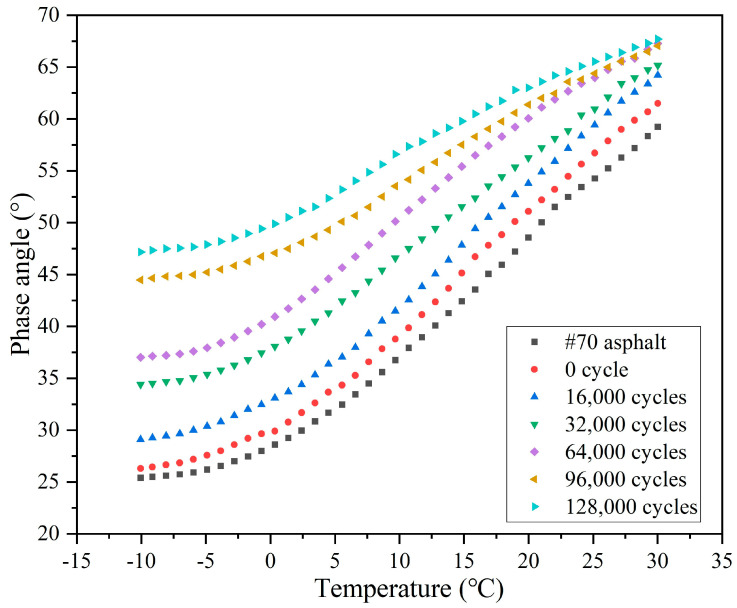
The δ of asphalt after different cycles of load.

The variations of the complex modulus (G*) and phase angle (δ) of the extracted asphalt with the number of load cycles are depicted in Figure 21 and Figure 22, respectively. Overall, the G* of asphalt presents a decreasing trend, while the δ shows an increasing trend with the test temperature, which is due to the molecules in the asphalt transforming from the frozen state into the flowing state owing to the elevated test temperature, and the asphalt changing from the elastic state to the viscous state, and the flow ability gradually increasing.

In comparison to the base 70 asphalt, the G*of the asphalt without external cyclic load decreases slightly and the phase angle increases slightly, as shown by the decrease in G* from 1.76 × 10^8^ to 1.61 × 10^8^ and the increase in δ from 25.4° to 26.3° at −10 °C. The premature release of a small quantity (4.5%) of sunflower oil from the capsules in the mixing and compaction period lead to a slight softening of the asphalt, which is evidenced by a reduction in G* and a rise in δ.

When the cyclic load is conducted on the beams, the G* of extracted asphalt decreases sharply and the phase angle increases significantly. Following 16,000 cycles of load, the G* at −10 °C decreases from 1.61 × 10^8^ to 9.84 × 10^7^ and the δ increases from 26.3° to 29.1°. During cyclic load, the capsules release sunflower oil (rejuvenator) into the asphalt concrete, thereby improving its flowability by reducing viscosity. As the number of load cycles rises, the G* decreases and the δ increases. When the load cycle increases from 16,000 to 128,000, the G* of asphalt at −10 °C decreases from 9.84 × 10^7^ to 3.68 × 10^7^ and the δ increases from 29.1° to 47.2°. With the increase in the load cycle, the rejuvenator release ratio of the capsules increases. More oil in the asphalt would endow it with a faster flow ability. Overall, the G* and δ decrease and increase with the rising of load cycle, which implies that the capsules gradually release inner encapsulated sunflower oil under the action of load stress.

#### 3.4.2. Rheological Property of Asphalt Binder Following Different Load Pressures

**Figure 23:**
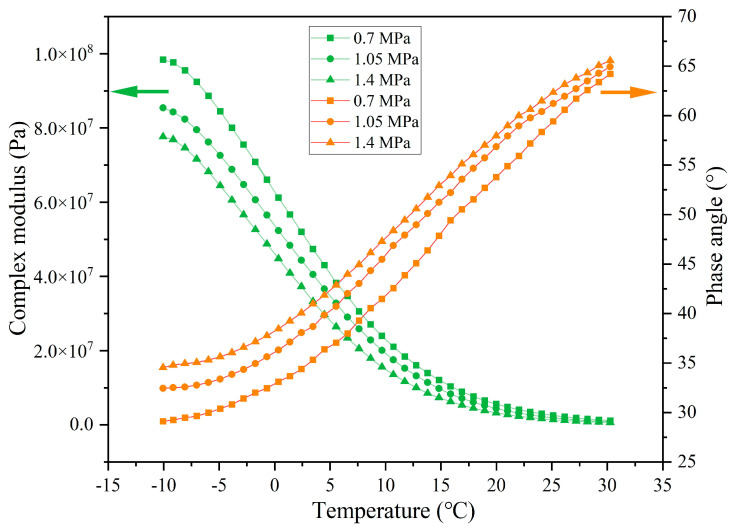
The rheological index of asphalt under 16,000 load cycles at different load pressures.

**Figure 24:**
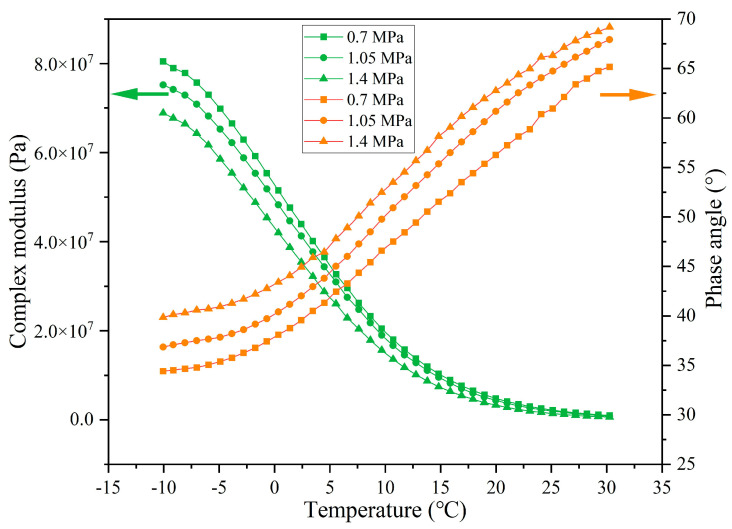
The rheological index of asphalt under 32,000 load cycles at different load pressures.

**Figure 25:**
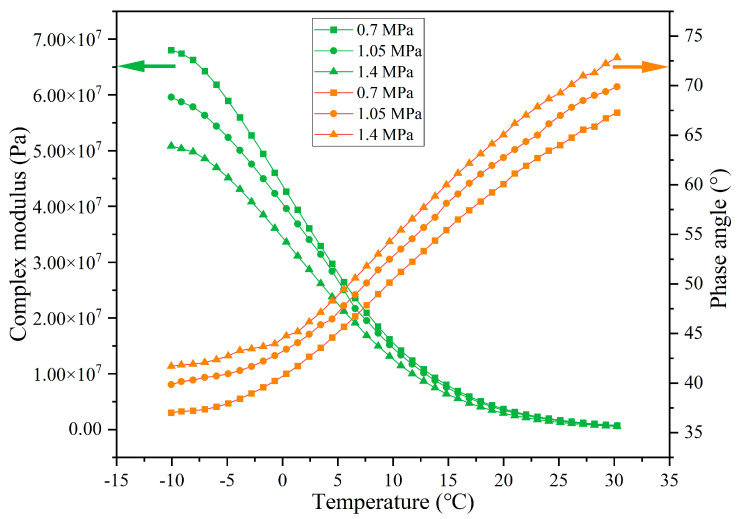
The rheological index of asphalt under 64,000 load cycles at different load pressures.

**Figure 26:**
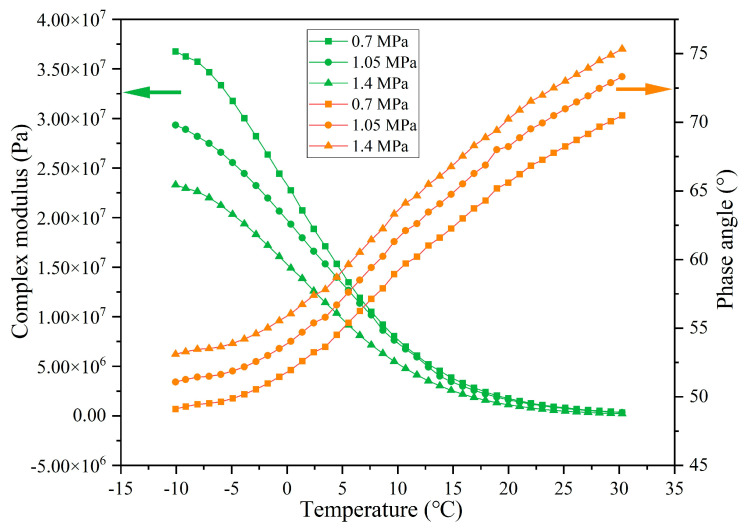
The rheological index of asphalt under 128,000 load cycles at different load pressures.

The rheological indexes of extracted asphalt under different load pressures are presented in Figure 23, Figure 24, Figure 25 and Figure 26. When experiencing the same cycles of load, with the increase in load intensity, the G* of asphalt shows a decreasing trend while the δ shows an increasing trend. For example, when the load cycles are 32,000, the G* of asphalt at −10 °C under load pressures of 0.7 MPa, 1.05 MPa, and 1.40 MPa is 8.05 × 10^7^, 7.52 × 10^7^, and 6.88 × 10^7^ and the δ is 34.4°, 36.8°, and 39.9°, respectively. Higher load pressure will make the capsules release more sunflower oil, and the released rejuvenator improves the flow ability of the asphalt. When the load pressure is 1.40 MPa, the G* of asphalt at −10 °C after 16,000, 32,000, 64,000, and 128,000 cycles of load is 7.76 × 10^7^, 6.89 × 10^7^, 5.08 × 10^7^, and 2.33 × 10^7^ and the δ is 34.5°, 39.9°, 41.7°, and 53.1°, respectively, which indicates that the flow ability of asphalt under heavy load conditions improves gradually with the increase in the number of loads, and this also shows that the capsules within asphalt concrete still maintain the characteristics of gradual release under heavy load conditions.

#### 3.4.3. Rheological Property of Asphalt Binder Under Different Load Application Frequencies

**Figure 27:**
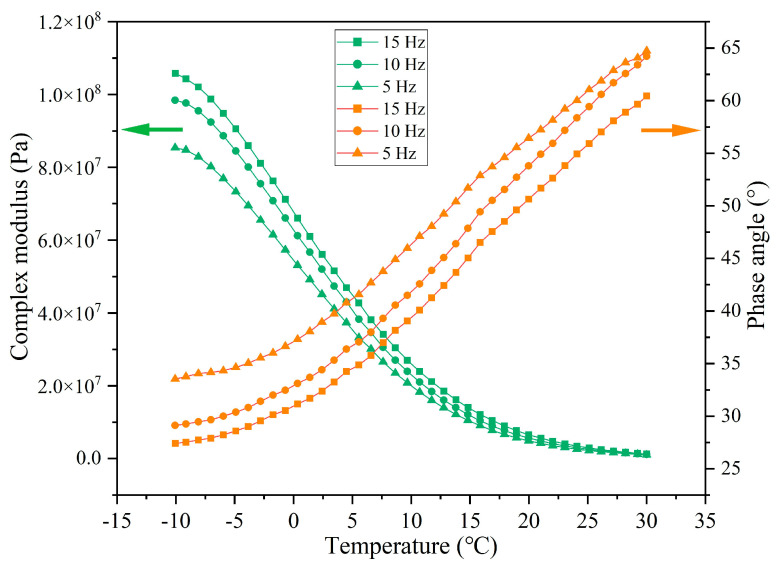
The rheological index of asphalt under 16,000 load cycles at different load frequencies.

**Figure 28:**
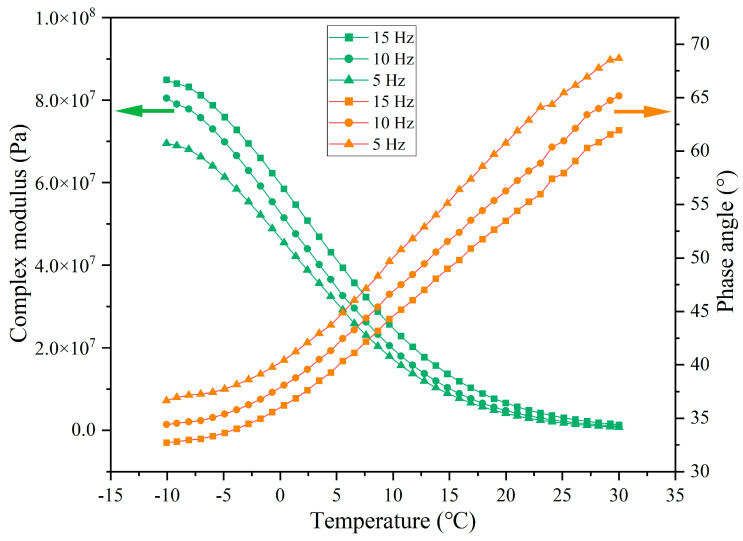
The rheological index of asphalt under 32,000 load cycles at different load frequencies.

**Figure 29:**
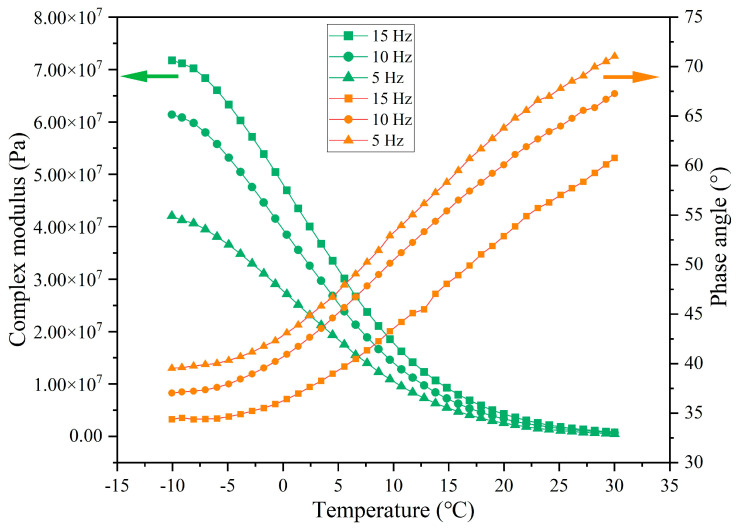
The rheological index of asphalt under 64,000 load cycles at different load frequencies.

**Figure 30:**
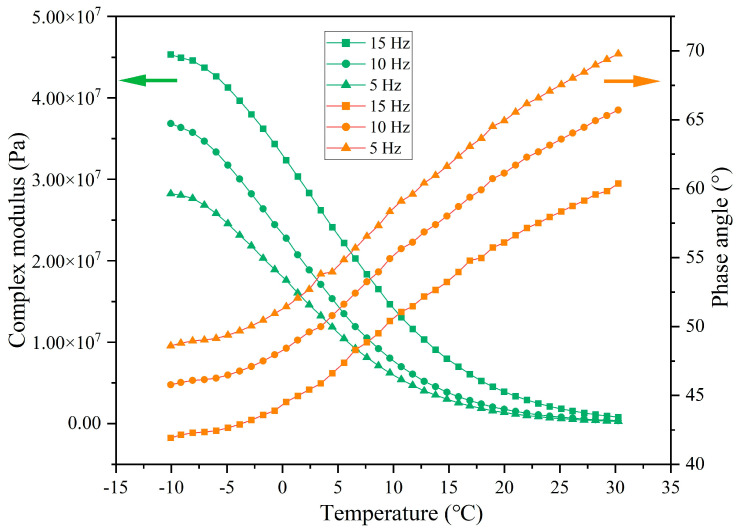
The rheological index of asphalt under 128,000 load cycles at different load frequencies.

The rheological indexes of extracted asphalt under different load application frequencies are presented in Figure 27, Figure 28, Figure 29 and Figure 30. When the number of loads is unchanged, as the load application frequency decreases, the G* of asphalt gradually decreases and the δ gradually increases. When the number of loads is 64,000, the G* of asphalt at −10 °C is 7.17 × 10^7^, 6.13 × 10^7^, and 4.20 × 10^7^ under the load application frequencies of 15 Hz, 10 Hz, and 5 Hz, respectively, and the phase angle is 34.4°, 37.1°, and 39.5°, respectively. When the load cycle is 128,000, the G* of asphalt at −10 °C is 4.53 × 10^7^, 3.68 × 10^7^, and 2.82 × 10^7^, respectively, and the δ is 41.9°, 45.7°, and 48.6°, respectively, at the load application frequencies of 15 Hz, 10 Hz, and 5 Hz. Under the fixed load condition, lower load frequency leads to longer single load action time, which means that the total stress action time on the capsules will improve and the released rejuvenator will increase.

### 3.5. SARA Fractions of Extracted Asphalt Binder After Cyclic Load

#### 3.5.1. SARA Fractions of Asphalt After Different Load Cycles

**Figure 31:**
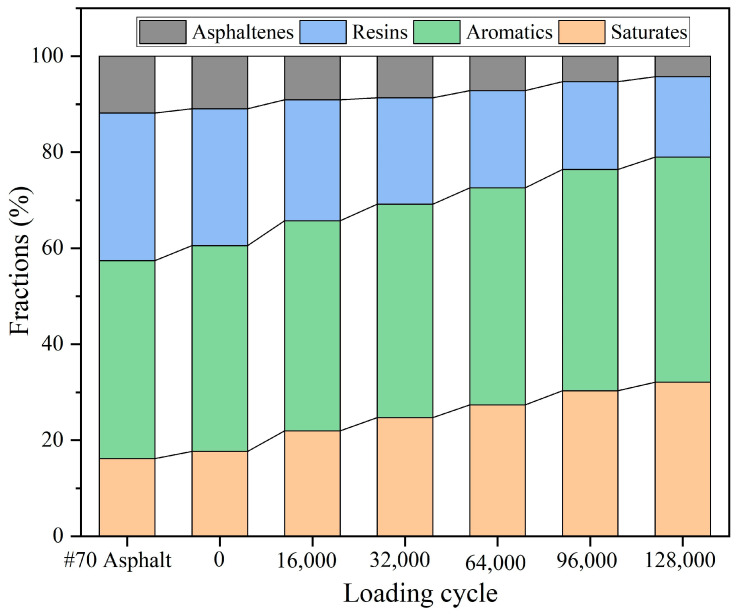
The SARA fractions of asphalt after different cycles of load.

The component content of the extracted asphalt after different load cycles is shown in Figure 31. In the base 70# asphalt, saturates, aromatics, resins, and asphaltenes account for 16.18%, 41.26%, 30.75%, and 11.81% of the composition, respectively. Prior to load (0 cycle), the relative contents of saturates, aromatics, resins, and asphaltenes are 17.68%, 42.93%, 28.54%, and 10.95%, respectively. This shift towards lighter components is attributed to the premature release of a small amount of sunflower oil from the capsules in the concrete preparation processes.

Overall, with the increase in the load cycles, the relative contents of saturate and aromatic fractions in asphalt both showed an increasing trend, and the relative contents of resins and asphaltenes showed a decreasing trend. When the number of loads increases from 0 to 128,000, the relative content of the saturate fraction increases from 17.68% to 32.08%, the relative content of the aromatic fraction increases from 42.93% to 46.91%, the relative content of the resin fraction decreases from 30.75% to 16.72%, and the relative content of asphaltene decreases from 11.81% to 4.29%. With the increase in load cycles, the capsules in the asphalt concrete gradually release the internal sunflower oil and flow to the asphalt, thus improving the relative content of light components and realizing the partial regeneration of asphalt.

#### 3.5.2. SARA Fractions of Extracted Asphalt Binder Under Different Load Pressures

**Figure 32:**
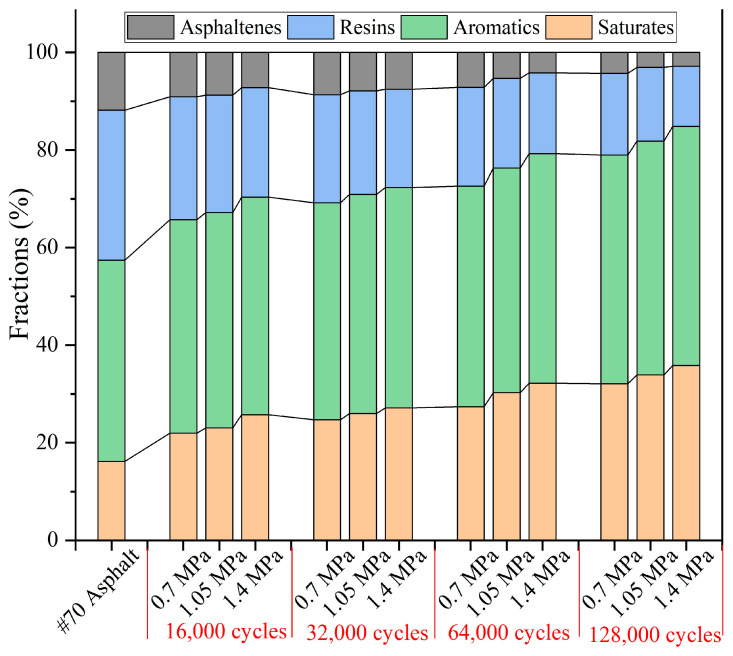
The SARA fractions of asphalt under different load pressures.

The component content of the extracted asphalt after different load pressures is shown in Figure 32. Overall, under the same number of loads, with the increase in load pressure, the relative contents of saturate and aromatic fractions both tended to increase, while the relative contents of resin and asphaltene tended to decrease. Under 16,000 cycles of load, with the load pressure improving from 0.7 MPa to 1.4 MPa, the relative content of saturates increases from 21.95% to 25.77%, the relative content of aromatics increases from 43.79% to 44.59%, the relative content of resins decreases from 25.19% to 22.45%, and the relative content of asphaltene decreases from 9.07% to 7.19%. Under 128,000 cycles of load, with the load pressure improving from 0.7 MPa to 1.4 MPa, the relative content of saturates increases from 32.08% to 35.84%, the relative content of aromatics increases from 46.91% to 49.02%, the relative content of resins decreases from 16.72% to 12.32%, and the relative content of asphaltene decreases from 4.29% to 2.82%. With increased load pressure under constant load cycles, the enhanced release of sunflower oil from the capsules correlated with a rise in the relative content of light components and a concurrent decrease in heavy components within the asphalt.

#### 3.5.3. SARA Fractions of Extracted Asphalt Binder After Different Load Frequencies

**Figure 33:**
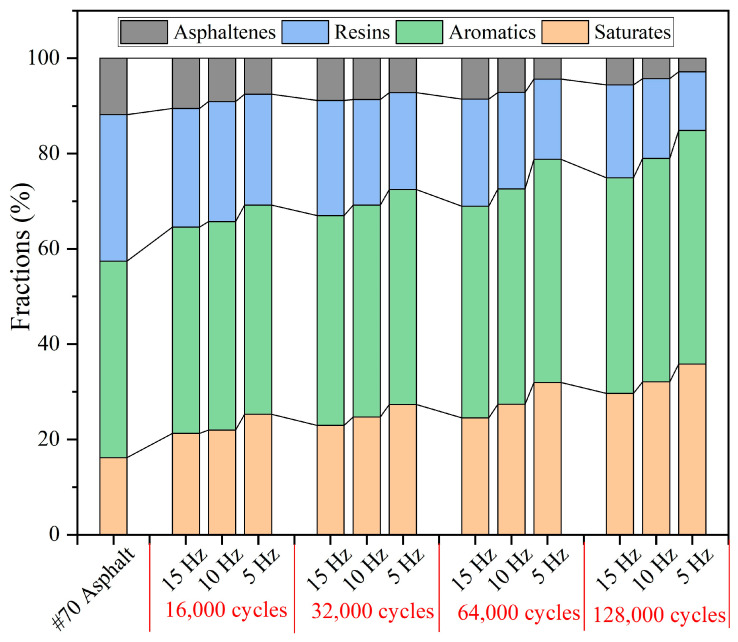
The SARA fractions of asphalt under different load frequencies.

The component content of extracted asphalt under various load frequencies is shown in Figure 33. Overall, under a fixed load cycle, as the load frequency decreased, the relative contents of saturates and aromatics increased, while the relative contents of resins and asphaltenes decreased. Under 16,000 cycles of load, with the load frequency decreasing from 15 Hz to 5 Hz, the relative content of saturates increases from 21.26% to 25.29%, the relative content of aromatics increases from 43.33% to 43.91%, the relative content of resins decreases from 24.87% to 23.29%, and the relative content of asphaltenes decreases from 10.54% to 7.51%. Under 128,000 cycles of load, with the load frequency decreasing from 15 Hz to 5 Hz, the relative content of saturates increases from 29.68% to 35.84%, the relative content of aromatics increases from 45.24% to 49.02%, the relative content of resins decreases from 19.49% to 12.32%, and the relative content of asphaltenes decreases from 5.59% to 2.82%. Under fixed cycles of load, as the load frequency decreases, the amount of sunflower oil released from the capsule increases; therefore the relative content of the light components in asphalt increases and the relative content of the heavy components decreases.

## 4. Conclusions

This study involves the fabrication of calcium–alginate composite capsules and the evaluation of their basic properties. The self-healing efficiency of test beams containing capsules is then assessed under various load conditions. Furthermore, the rejuvenator release ratios following different cyclic load intervals are determined. Finally, the rheological properties and chemical composition of the extracted asphalt binder are analyzed to elucidate the healing mechanism. The principal conclusions of this work are summarized below:

(1) The healing ratio of normal asphalt concrete exhibits minimal fluctuations with the load cycle, pressure, and frequency. The healing ratio of capsule-modified asphalt concrete increases as the load cycle and intensity increase and decreases as the load frequency increases. Overloading and speeding accelerate crack formation in asphalt pavements; the incorporation of capsules may mitigate this phenomenon.
(2) The rejuvenator in the capsules shows a gradual release feature. After 16,000, 32,000, 48,000, 64,000, 96,000, and 128,000 cycles of load (0.7 MPa), the rejuvenator release ratios of the capsules are 23.9%, 36.4%, 44.8%, 49.5%, 56.7%, and 60.3%, respectively. The long-lasting sustained release characteristics of the capsules support the realization of sustainable healing for asphalt pavement. Under fixed load cycles, the rejuvenator release ratio of the capsules increases with the increase in load pressure and decreases with the increase in load frequency. Overloading and speeding accelerate the release of rejuvenating agents, which may expedite crack closure and repair, thereby mitigating damage to asphalt pavements under severe traffic loading conditions.
(3) The released rejuvenator modifies the rheological properties of asphalt. The complex modulus and phase angle of asphalt decline and rise, respectively, with the increase in the rejuvenator release ratio. Meanwhile, the released rejuvenator can balance the component content of asphalt and improve the light components content of the asphalt, which realizes the regeneration of aged asphalt. The incorporation of capsules delays the aging of asphalt pavements, promising to extend their service life and enhance the resilience of road infrastructure.

This paper provides theoretical support for the service life of capsules under various traffic load conditions, facilitating their practical application and promotion in road engineering projects. During the real-service phase of asphalt pavement, the asphalt component is prone to aging under the synergistic effect of natural conditions and traffic-related influences. It should be noted that this study only focused its attention on the self-healing performance of fresh asphalt concrete that contains capsules under applied cyclic load. Given that the primary function of calcium alginate capsules is to replenish light components and restore aged asphalt, future research will focus on evaluating the healing efficacy in asphalt concrete that has undergone various aging treatments. Additionally, future research will focus on monitoring the in-service performance and healing effects of the capsules in practical engineering applications.

## Figures and Tables

**Table 1:** Basic information of raw materials.

Raw Material	Purity	Manufacturer
Sodium alginate	CP	Sinopharm Chemical Reagent (Beijing, China)
Anhydrous calcium chloride	CP	Sinopharm Chemical Reagent (Beijing, China)
Nano-Fe_3_O_4_ (50 nm)	99.9%	Chaowei nanomaterials Co., Ltd., Shanghai, China.
Sunflower oil	Food grade	Arowana Group Co., Ltd., Hangzhou, China.
Tween 80	AR	Sinopharm Chemical Reagent (Beijing, China)

**Table 2:** Basic information of sunflower oil.

Item	Value
Appearance	Light yellow liquid
Density (15 °C)	0.935 g/cm^3^
Viscosity (60 °C)	0.285 Pa·s
Flash point	230 °C

**Table 3:** Medium traffic class pavement design parameters.

Design Parameter	Value
Service life of pavement (years)	15
Traffic volume cumulative equivalent axle times (times/lane)	1 × 10^7^
Lane coefficient	0.4
Distribution coefficient of wheel grinding	0.5
Equivalent car axle load conversion factor	0.12

**Table 4:** Setting parameters of cyclic load under different vehicle speeds.

Speed (km/h)	Load Action Frequency (Hz)	Load Action Time (ms)	Rest Time (ms)	Load Cycle Time (ms)
30	5	200	800	1000
60	10	100	900	1000
90	15	66.67	933.33	1000

**Table 5:** The fundamental properties of Ca-alginate capsules [43].

Performance	Value
Average diameter (mm)	1.8
Rejuvenator content (%_mass_)	58.1%
Compressive strength (N)	11.8
Mass loss (%) at	
160 °C	2.8
200 °C	3.9

## Data Availability

The original contributions presented in this study are included in the article. Further inquiries can be directed to the corresponding authors.
